# Moisture-Driven Degradation Pathways in Prussian White Cathode Material for Sodium-Ion Batteries

**DOI:** 10.1021/acsami.0c22032

**Published:** 2021-02-18

**Authors:** Dickson O. Ojwang, Mikael Svensson, Christian Njel, Ronnie Mogensen, Ashok S. Menon, Tore Ericsson, Lennart Häggström, Julia Maibach, William R. Brant

**Affiliations:** † Department of Chemistry—Ångström Laboratory, Ångström Advanced Battery Centre, Uppsala University, Box 538, SE-751 21 Uppsala, Sweden; ‡ Institute for Applied Materials (IAM) and Karlsruhe Nano Micro Facility (KNMF), Karlsruhe Institute of Technology (KIT) Hermann-von-Helmholtz-Platz 1, 76344 Eggenstein-Leopoldshafen, Germany

**Keywords:** moisture sensitivity, sodium-ion batteries, Prussian white cathode, capacity degradation mechanisms, relative humidity

## Abstract

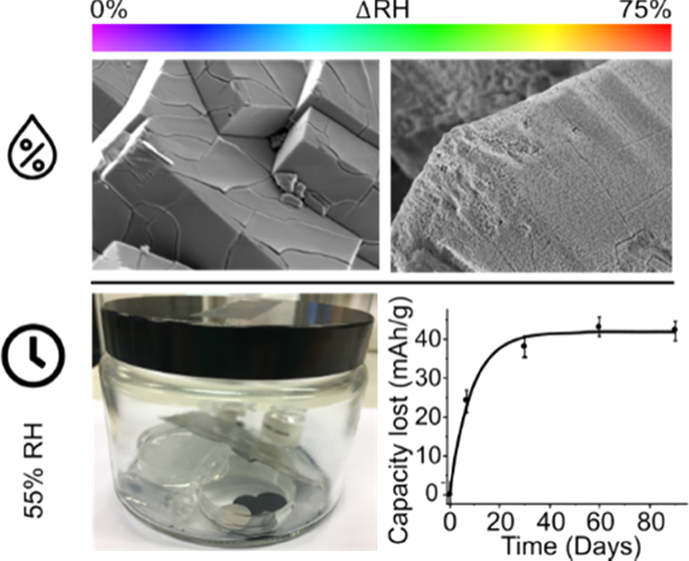

The high-theoretical-capacity (∼170 mAh/g) Prussian white (PW), Na_*x*_Fe[Fe(CN)_6_]_*y*_·*n*H_2_O, is one of the most promising candidates for Na-ion batteries on the cusp of commercialization. However, it has limitations such as high variability of reported stable practical capacity and cycling stability. A key factor that has been identified to affect the performance of PW is water content in the structure. However, the impact of airborne moisture exposure on the electrochemical performance of PW and the chemical mechanisms leading to performance decay have not yet been explored. Herein, we for the first time systematically studied the influence of humidity on the structural and electrochemical properties of monoclinic hydrated (M-PW) and rhombohedral dehydrated (R-PW) Prussian white. It is identified that moisture-driven capacity fading proceeds via two steps, first by sodium from the bulk material reacting with moisture at the surface to form sodium hydroxide and partial oxidation of Fe^2+^ to Fe^3+^. The sodium hydroxide creates a basic environment at the surface of the PW particles, leading to decomposition to Na_4_[Fe(CN)_6_] and iron oxides. Although the first process leads to loss of capacity, which can be reversed, the second stage of degradation is irreversible. Over time, both processes lead to the formation of a passivating surface layer, which prevents both reversible and irreversible capacity losses. This study thus presents a significant step toward understanding the large performance variations presented in the literature for PW. From this study, strategies aimed at limiting moisture-driven degradation can be designed and their efficacy assessed.

## Introduction

Lithium-ion batteries (LIBs) have been instrumental in enabling our portable society due to their superior specific energy density, energy efficiency, and long cycle life.^1^ Today they are set to support the energy revolution powering electric and low emission plug-in hybrid vehicles in addition to stationary energy storage applications.^2^

However, most easily accessible lithium reserves are in either geographically remote or politically sensitive areas. The low cost, environmental benignity, and natural abundance of sodium resources have made Na-ion batteries (NIBs) a competitive alternative to their lithium counterparts for large-scale electric energy storage (EES) systems. To enable the advancement of Na-ion technology, electrode materials based on transition-metal oxides, organic compounds, and polyanionic compounds based on phosphates or sulfates have been investigated as insertion electrodes.^3−5^ However, they usually suffer from insufficient cycling and rate performance mainly from structural instability caused by multiple phase transitions and substantial volume changes during cycling.^6^ This has led to intensive investigation of Prussian blue analogues PBA-*A*_*x*_*M*[*M*′(CN)_6_]_1–*y*_·*nG* (where *A* is charged guest species, *M* and *M*′ are transition-metal cations, *y* is the amount of [*M*(CN)_6_] vacancies, and *G* is a neutral guest species) because of their open-framework structure, facile synthesis, nontoxicity, and tunable composition. In the literature, most Prussian blue analogues (PBAs) exist with a range of different compositions in addition to variations of *x, y*, and *n* contents, leading to highly variable electrochemical performance even for the same compositional family.^7−10^ Further variability originates from a wide spectrum of synthesis routes, evaluation procedures, and storage conditions.^11^ In particular, the electrochemical performance is broadly accepted to be heavily affected by the presence of water. For instance, Goodenough et al. reported improved cycling stability and a high reversible capacity when the pristine monoclinic phase of Na_*x*_*M*[Fe(CN)_6_](*M* = Mn, Fe) Prussian white (PW) was converted to a rhombohedral structure through dehydration.^7,9^ While crystal water is known to be crucial in facilitating good electrochemical performance of other electrode materials such as Birnessite MnO_2_, FePO_4_·*x*H_2_O, and WO_3_·2H_2_O,^12−15^ PW is negatively affected by water. Namely, its presence increases the potential of the low-spin iron plateau above the oxidative stability limit of water (3.94 V vs Na/Na^+^), leading to irreversible gas formation, structural collapse, capacity degradation, and poor cycle lifetime.^16,17^ All of these detrimental effects will impede the battery’s practical application.^8−10,18,19^

Consequently, thorough drying prior to incorporation in a nonaqueous electrochemical cell is strongly recommended. However, PBAs are known to behave as molecular sponges, exhibiting affinity for flue and natural gases such as CO_2_, N_2_, NO, H_2_S, CH_4_, and SO_2_,^20−22^ in addition to a strong affinity for water uptake.^23−25^ As a result, regardless of drying procedures, there are justifiable concerns that the material performance will be altered depending on the conditions under which it is processed, handled, and stored. Previously, work has been carried out on the humidity-induced magnetism of Co_*x*_^II^Mn_1–*x*_^II^[Cr^III^(CN)_6_]_2/3_·*z*H_2_O-PBA under controlled relative humidity (RH) conditions.^26^ More recently, there was an investigation on protecting Na_*x*_Fe[Fe(CN)_6_] from the readsorption of water through surface passivation.^27^ Assessing the actual effectiveness of such strategies is only possible if the underlying mechanism for and severity of the degradation is known. However, a systematic investigation of the mechanism behind water-driven structural degradation of a cation-rich, vacancy-poor PW has not been performed. A model describing the chemical processes occurring on exposure to moisture will enable informed strategies to be developed, mitigating degradation during handling and storage as well as guaranteeing practical implementation of PW-based batteries.

In this study, we address the question of moisture sensitivity for the Fe-based PW Na_1.80(5)_Fe[Fe(CN)_6_]·1.84(3)H_2_O. Fe-PW is particularly attractive for commercialization compared to other PBAs as it has the best performance-to-cost ratio.^7,8,28^ Thus, it is a prime candidate for investigating the mechanism of how different RHs and storage times change the properties. The effect of moisture was studied for the monoclinic hydrated (M-PW) and rhombohedral dehydrated (R-PW) phase first as a function of RH level and then as a function of time for 55% RH (Figure S1). By analyzing bulk, surface, and electrochemical performance changes, the chemical origins of reversible and irreversible sodium loss are explained.

## Experimental Section

### Synthesis

The synthesis of PW Na_1.80(5)_Fe[Fe(CN)_6_]·1.84(3)H_2_O proceeded by preparing a solution of Na_4_[Fe(CN)_6_]·10H_2_O (Sisco Research Laboratories Pvt. Ltd.; extra pure AR, 99%) in deoxygenated water and heating to 80 °C. To the heated solution, a substoichiometric amount of HCl (37%) was added over 5 h using a syringe pump to produce the acid-facilitated self-decomposition of Na_4_[Fe(CN)_6_]·10H_2_O. All the while, N_2_ was flowing during the entire reaction process that lasted 24 h. The obtained precipitate was filtered, washed three times with deionized water, and finally dried at 80 °C under vacuum overnight. The resulting powder was stored in a desiccator for further studies.

### Humidity Control

Prior to humidity-dependent studies, the as-prepared Na_1.80(5)_Fe[Fe(CN)_6_]·1.84(3)H_2_O powder sample was treated under two conditions: (1) dried under Ar at 100 °C for 24 h (M-PW) and (2) dried under vacuum at 200 °C for 24 h (R-PW). A few milligrams of the dried M-PW and R-PW sample powders were placed in different sealed containers under Ar with humidity controlled using saturated aqueous salt solutions of alkali metals (Table S1). The humidity of each sample holder was measured by a humidity meter (Fisherbrand Certified Traceable digital hygrometer). The sample powders were taken out for characterization after 7 days.

### Material Characterization

Thermogravimetric analysis (TGA) was performed on a TGA-Q500 thermogravimetric analyzer (TA Instruments) from room temperature to 500 °C at a heating rate of 5 °C/min under flowing N_2_. Powder X-ray diffraction (XRD) patterns were collected in Debye–Scherrer mode on a STOE Stadi P diffractometer, using monochromated (Ge) Cu Kα radiation (λ = 1.5406 Å) with a Dectris Mythen 1K position-sensitive detector, operating at 45 kV and 40 mA. All samples were sealed in 0.5 mm borosilicate capillaries in an Ar-filled glovebox prior to measurements. Pawley fits and Rietveld refinements^29^ of the powder patterns were carried out using the TOPAS software package.^30^ The zero error of the diffractometer was calibrated using a NIST 640b Si standard. Sample morphologies were observed using a scanning electron microscope (LEO-1530). Mössbauer spectra were recorded at room temperature on a spectrometer with a constant acceleration-type oscillator and a ^57^CoRh source. The M-PW samples kept at different RHs were mixed with BN under Ar and sealed in aluminum pouches. The resulting absorbers had concentrations of ∼10 mg/cm^2^. Calibration spectra were recorded using natural Fe metal foil as a reference absorber. The final spectra were folded and fitted using the least-squares Mössbauer fitting program Recoil to obtain the values of the isomer shift (δ), electric quadrupole splitting (Δ), full width at half-maxima (Γ) of the Lorentzian absorption lines, and spectral intensities I.

Raman spectra were acquired using a Renishaw Raman microscope equipped with a 532 nm diode laser and a grating of 1800 l/m. Fourier transform infrared (FTIR) spectra were recorded on a Spectrum One attenuated total reflection (ATR)-FTIR spectrometer in the range of 4000–400 cm^–1^. The signal was obtained by averaging 40 scans at a spectral resolution of 4 cm^–1^. The elemental ratio of Na and Fe was determined by inductively coupled plasma optical emission spectrometry (ICP-OES), and CNH by elemental analysis. The ICP-OES and elemental analyses were performed by Medac Ltd., U.K.

X-ray photoelectron spectroscopy (XPS) measurements were performed on a Thermo Scientific K-alpha spectrometer using monochromatized Al Kα radiation (1486.6 eV, 400 μm spot size). The photoelectrons were detected by a concentric hemispherical analyzer with a pass energy of 50 eV. Prior to the measurements, the samples were prepared in an Ar-filled glovebox, where cells were opened and rinsed with dimethyl carbonate (DMC). The spectral fitting was done with one or more Voigt profiles (binding energy uncertainty: ±0.2 eV), and Scofield sensitivity factors were applied for quantification using the Avantage software package.^31^ All spectra were referenced to the C 1s peak (C–C, C–H) at a binding energy of 285.0 eV controlled by means of the photoelectron peaks of metallic Cu 2p_3/2_, Ag 3d_5/2_, and Au 4f_7/2_. To check for sample degradation during the measurements, repeated carbon (C 1s) spectra were recorded at the beginning and end of each resolution analysis.

### Electrochemical Testing

The positive electrode was prepared by casting a slurry consisting of 80 wt % PW, 10 wt % carbon black (C-NERGY SUPER C65), and 10 wt % poly(vinylidene fluoride) (PVDF, Solef 5130) binder onto Al foil. Upon predrying at 60 °C under vacuum for 1 h, circular electrodes were punched out and introduced into an Ar-filled glovebox. Prior to cell assembly, the electrodes were divided into two parts and further dried for 24 h at: (1) 100 °C under Ar (M-PW) and (2) 140 °C under vacuum (R-PW). A few dried M-PW and R-PW electrode slices were separated and kept at 55% RH for 7, 30, 60, and 90 days. These electrodes were dried again at 100 °C under Ar for M-PW and 140 °C under vacuum for the R-PW samples before being assembled into full cells. The electrolyte was 1 M NaPF_6_ (Stella) dissolved in ethylene carbonate/diethyl carbonate (EC/DEC, 1:1 v/v) solution and a glass fiber and a partially charged PW were used as the separator and counter electrode, respectively. Galvanostatic (i.e., constant current) tests were performed at room temperature between 2.6 and 3.8 V (vs Na/Na^+^) at 0.1 C on a LAND CT2001A battery tester.

## Results

**Figure 1:**
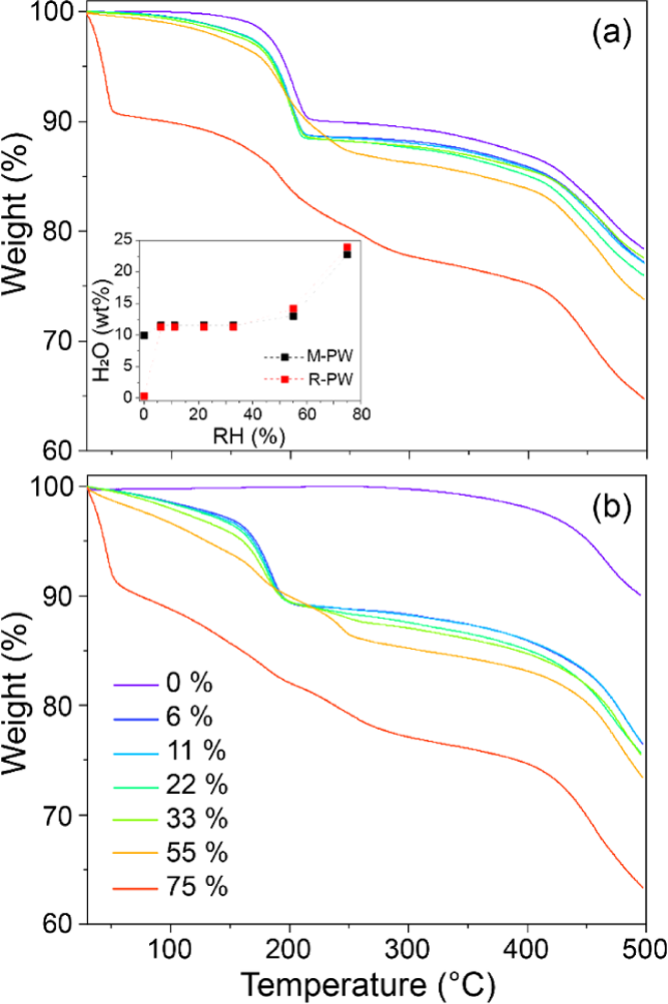
TGA curves of (a) M-PW and (b) R-PW samples recorded from 30 to 500 °C at a scan rate of 5 °C/min under N_2_ flow. The inset shows the estimated water content vs RH measured between 30 and 300 °C.

From ICP-OES, it was determined that the nominal Na/Fe ratio was roughly 1.08(3):1, indicating an excess of sodium. This excess sodium was found to originate from NaCl, which was present as an impurity phase using XRD (at ∼31.7°). TGA measurements of pristine M-PW show a weight loss of ∼9.9 wt % when *T* < 200 °C, corresponding to the release of both surface-adsorbed and interstitial water (Figure 1a). By comparison, the R-PW is thermally stable to ∼300 °C with a negligible weight loss, ∼0.3 wt % (Figure 1b). Thus, the initial compositions can be described as Na_1.80(5)_Fe[Fe(CN)_6_]_0.95(3)_·*n*H_2_O + 0.20(1)NaCl, where *n* = 1.84(3) for M-PW and 0.05(1) for R-PW. Here, the molar ratio and associated error for NaCl were determined from Rietveld refinement of a two-phase model against XRD data (Figure S3 and Tables S4 and S5).

### Degradation as a Function of Relative Humidity

Initially, the total uptake of water by each material was estimated by TGA. Both M-PW and R-PW exhibit highly similar mass loss profiles after exposure to moisture equal to and greater than a relative humidity level of 6%. This can be seen also by comparing the mass loss measured between 30 and 300 °C presented in the inset of Figure 1. Mass losses in this region can be largely attributed to water only.^32^

**Figure 2:**
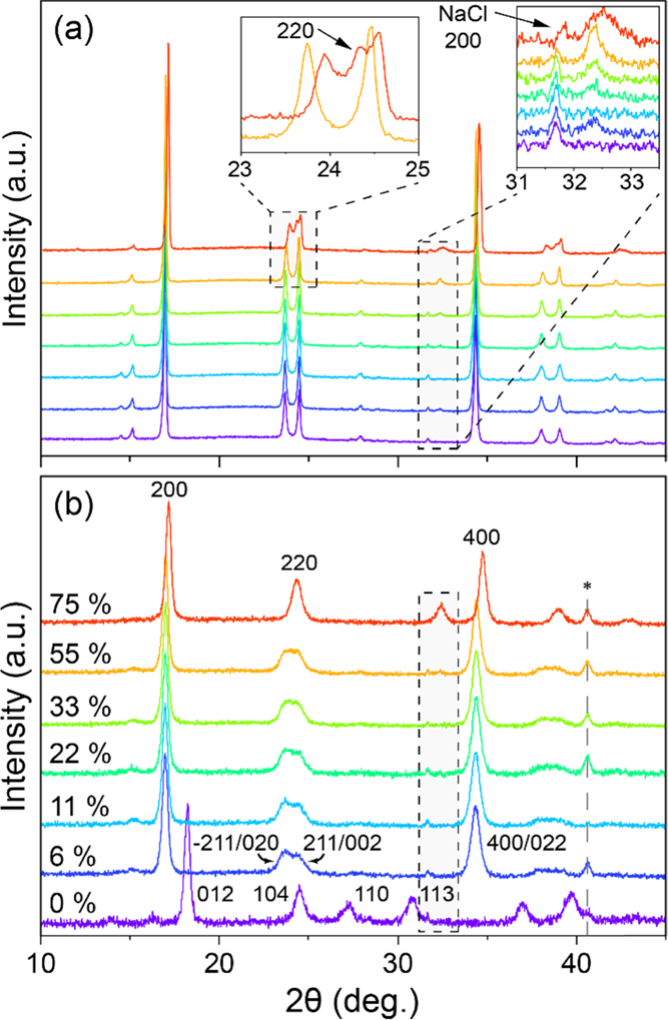
Powder XRD (PXRD) patterns of (a) M-PW and (b) R-PW samples at different RHs. The shaded areas mark the region where the NaCl impurity can be seen and where the Na_4_[Fe(CN)_6_] precursor appears.^36^ The * in (b) indicates an additional reflection originating from the collimation slits implemented on the diffractometer during these measurements.

To evaluate the structural changes of the samples under different RH values, XRD patterns were recorded after 7 days under each humidity condition. The M-PW sample (Figure 2a) maintains a monoclinic structure (**P**21/*n*) from 0% ≤ RH ≤ 55% albeit with a slight reduction of the unit cell volume (Figure S4), while at 75% RH, growth of a cubic phase with a ∼3% reduction in volume is indicated by the appearance of the 220 reflection. These changes imply the loss of sodium from the bulk structure (Figure 2a). In comparison, the R-PW immediately converts from *R*3̅ to **P**21/**n** on exposure to 6% RH, as evidenced, for example, by the emergence of the 200/011, −211/020, 211/002, and 400/022 reflections (Figure 2b).^7,11^ After converting from *R*3̅ to **P**21/*n*, this phase is maintained until 55% RH, but transforms to cubic under 75% RH.^8,9,32−34^ Compared with M-PW, the unit cell volume is seen to decrease to a greater extent for all RH% levels, implying a more significant loss of bulk sodium combined with oxidation of Fe^2+^ to Fe^3+^ due to reaction with water.^35^ In both R-PW and M-PW, the NaCl impurity remains unchanged at ∼31.7° and a new peak at ∼32.5° corresponding to Na_4_[Fe(CN)_6_] grows for increasing RH above 22% (Figure 2a, inset). Full XRD datasets and Pawley fits used to obtain the unit cell volumes are given in the SI. To further explore the loss of bulk sodium from the structure, Mössbauer spectroscopy measurements were performed.

**Figure 3:**
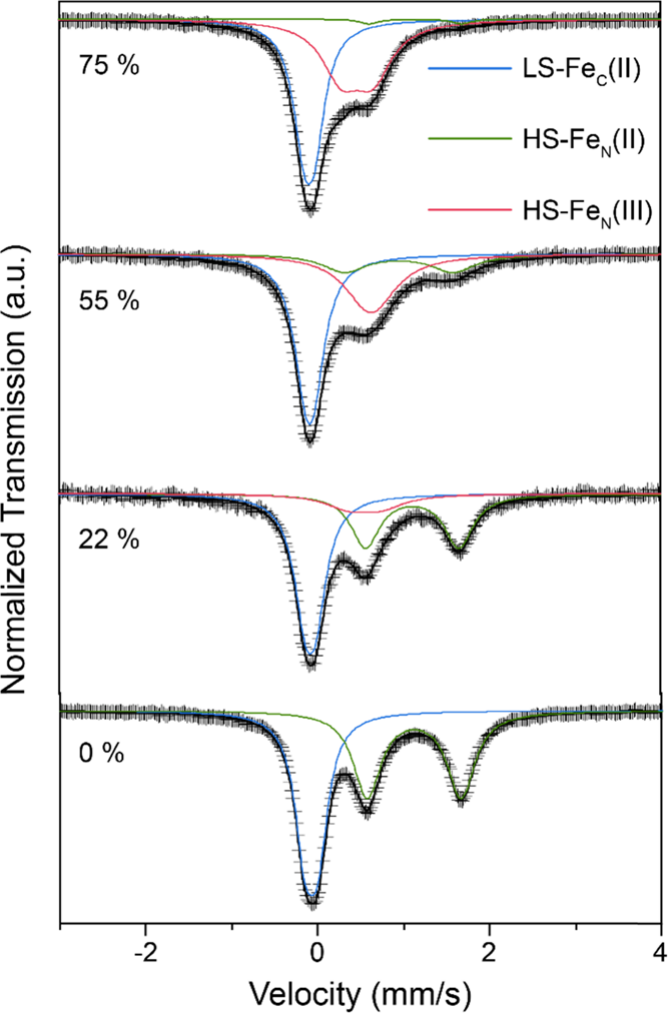
Representative Mössbauer spectra of M-PW sample at 295 K under different RH values.

The Mössbauer spectrum of M-PW at 295 K under 0% RH shows the well-known signature of monoclinic PW with Na content >1.8 and only two iron environments, i.e., a doublet with larger quadrupole splitting for HS-Fe^2+^N_6_ octahedra and a doublet with smaller quadrupole splitting for LS-Fe^2+^C_6_ octahedra, accordingly denoted as HS-Fe_N_ and LS-Fe_C_ (Figure 3).^11,32^ The Mössbauer parameters derived from fitting the spectra shown in Figure 3 are summarized in Table S6 in the SI.

Under 22% RH, a new component characterized by a small unresolved doublet arises and is ascribed to the presence of HS-Fe^3+^N_6_. An increase in the RH values to 55 and 75% RH leads to an increase in the intensity of the HS-Fe^3+^N_6_ doublet that correlates with a decrease in the HS-Fe^2+^N_6_ signal. The relative change in the sodium content with increasing RH values has been estimated based on the isomer shift (δ) values from our earlier studies^11,32^ (Figure S5) and in this study (Table S6). The results indicate a faster drop in the sodium content at low and high RH values (Figure S6). However, the overall trend agrees with the trends in water uptake (Figure 1) and cell parameters (Figure S4). In general, the Mössbauer data confirm the oxidation of iron for increasing humidity levels.

**Figure 4:**
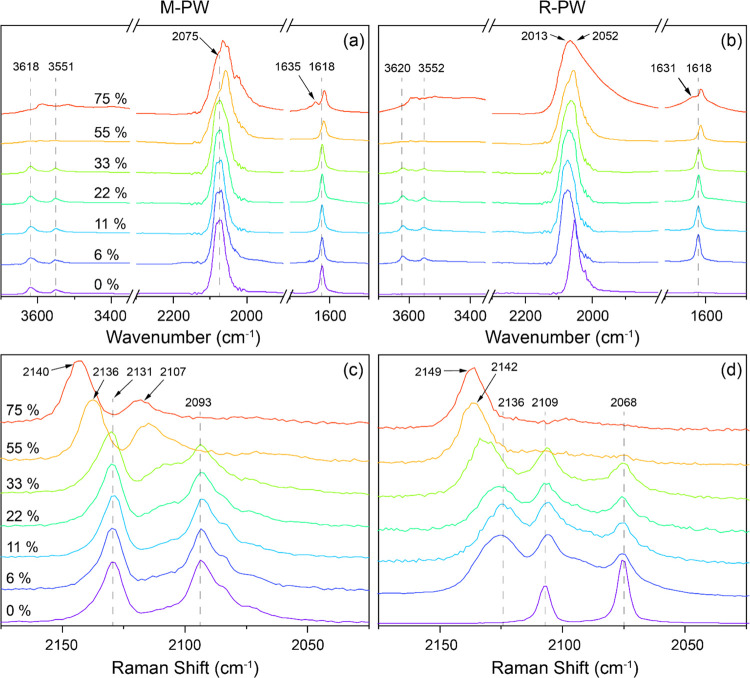
IR spectra of (a) M-PW and (b) R-PW, and Raman spectra of (c) M-PW and (d) R-PW.

Additional information on the uptake of water and changing oxidation state of iron for higher humidity levels was extracted from IR and Raman measurements. The IR spectra of M-PW and R-PW shown in Figure 4a,b reveal a strong Fe^2+^-ν(CN) band at ∼2075 cm^–1^.^37^ Deconvolution of this band revealed the presence of additional bands (Tables S7–S10). The corresponding Raman modes, E_g_ and A_1g_, are centered at 2130 and 2093 cm^–1^, respectively.^38^ Shifts in the ν(CN) band position, broadening, and variation in its relative intensities strongly suggest changes in the average valence state of iron.^39^

The sharp absorption bands seen around 3618 and 1618 cm^–1^ correspond to O–H stretching and H–O–H bending modes. The weak and broad absorption bands at ∼3646 and ∼3396 cm^–1^ are, respectively, assigned to non-hydrogen-bonded H_2_O or hydroxide and hydrogen-bonded H_2_O.^40−42^ The broadening of H–O–H band and the emergence of a new band at ∼1635 cm^–1^ under 75% RH can be due to interactions of additional water layers at the surface, that is, water interacting primarily with other water molecules (Figure S7). No O–H and H–O–H bands were observed for R-PW under 0% RH (Figure 4b), alluding to a completely dehydrated structure.

Summarizing the above measurements, as water is absorbed onto the material, sodium is lost from the bulk structure and, eventually, Na_4_[Fe(CN)_6_] is formed. However, the mechanism connecting these processes is not yet clear. Thus, SEM and XPS were implemented to search for surface deposits explaining the lost sodium.

**Figure 5:**
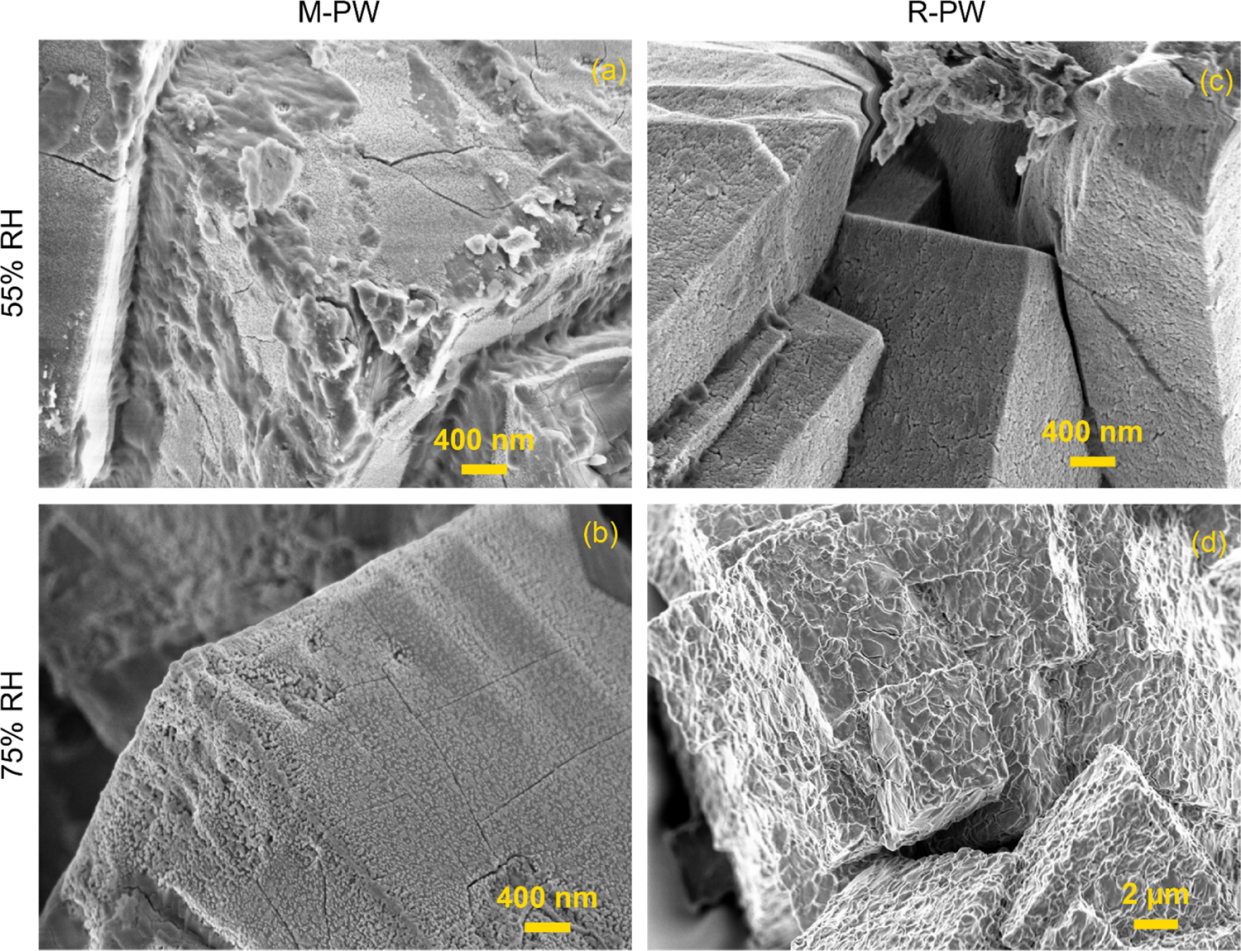
Representative SEM images of (a, b) M-PW at 55 and 75% RH and (c, d) R-PW at 55 and 75% RH obtained using an accelerating voltage of 2 kV. Note: Similar morphological features are seen in the range between 0 and 33% RH and are provided in the SI.

SEM micrographs shown in Figure 5 reveal cubic morphologies and an average particle size of ∼6 μm with RH-dependent surface texture for both samples. For M-PW, smooth surfaces that crack on high magnification due to localized heating and dehydration are evident at 0% RH (Figure S8). This appearance was consistent for the samples exposed to 6–33% RH. Once 55% RH was reached, selected regions show increased surface roughness (Figure 5a), and at 75% RH, it appears as if a precipitate has grown on the surface of the cubes (Figure 5b). Similar features are seen also for R-PW; however, a thin surface deposit starts to appear at 55% RH, while severe surface exfoliation occurs at 75% RH, implying that growth of the precipitates does not take place directly at the surface but from within cracks that are formed due to the volume contraction during the initial water removal. As revealed by XRD data, the initial drying of M-PW to R-PW leads to large structural strain due to severe lattice distortion and volume change of ∼19%. Subsequently, on rehydration, this phase transition is reversed and the volume expands again by ∼19%; however, the peaks remain quite broad, indicating either the presence of residual strain or finer cracks, which are not directly observed at this magnification level in the SEM. This initial phase transition (during dehydration and rehydration) damages the single crystal particles providing increased access to water, enhancing the damage on further moisture exposure (Figure 5c,d).

**Figure 6:**
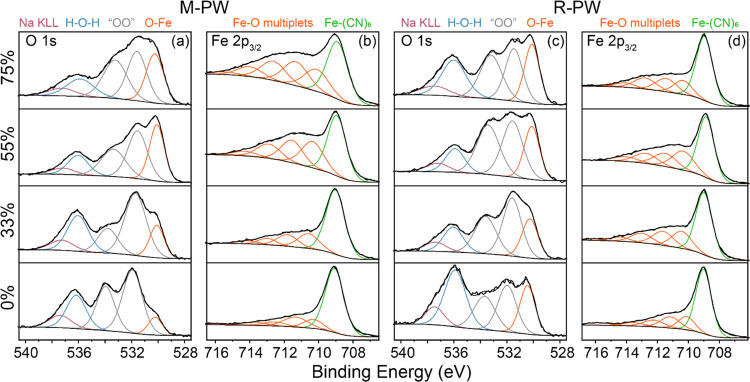
O 1s and Fe 2p_3/2_ XPS spectra of (a, b) M-PW and (c, d) R-PW samples at different RHs. “OO” refers to other oxides.

To determine the chemical composition of the surface deposits, Fe 2p_3/2_ and O 1s XPS spectra were recorded from M-PW and R-PW powders exposed to 0% RH, 33% RH, 55% RH, and 75% RH. The O 1s and Fe 2p_3/2_ spectra shown in Figure 6 are discussed. The O 1s spectra gather signals coming from all oxygenated species at the surface, including Na KLL at 537.8 eV, the peak around 530 eV (orange) corresponding to the O–Fe environment, and the blue peak around 536 eV is assigned to oxygen from water. Two additional peaks are detected at 531.5 and 533.6 eV, respectively. These peaks called “other oxides” could be attributed to oxygenated species such as NaOH, carbon oxides, and NO*_x_*. Most notable from the O 1s spectra, however, is the growth of O–Fe for increasing RH for both M-PW and R-PW. To determine the nature of these iron species, the Fe 2p_3/2_ components of the Fe 2p spectra were fitted. The main 2p_3/2_ component (green) located around 709 eV can be assigned to Fe–(CN)_6_.^43,44^ In the 716–709 eV binding energy range, five components (orange) are attributed to iron oxide multiplets. These multiplets are a signature of ferrous (Fe^2+^)- or ferric (Fe^3+^)-type oxides or hydroxides.^44^ A comparison of %(O–Fe) to %(Fe–O) from the O 1s and Fe 2p spectra, respectively (Figure S9), reveals that iron at the surface is in the +2 state for the pristine samples; however, the deposits that grow on exposure to 55 and 75% RHs are closer to +3. Interestingly, the growth of Fe–O species appears to be reduced for the R-PW sample, which is in contrast to the bulk XRD measurements (Figure S3). This may be due to the increased crystallite cracking observed for R-PW and growth of degradation products deeper inside the material, which are thus not directly probed by XPS. The exfoliation observed in Figure 5d for R-PW suggests this may be the case.

Summarizing the results obtained as a function of relative humidity level, it was seen that M-PW and R-PW lose sodium from the bulk structure and correspondingly experience oxidation of iron. Given that the water source implemented was not deoxygenated, a possible redox process to explain this would be as follows
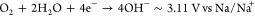
1
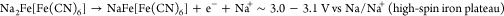
2
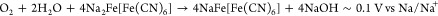
3The sodium hydroxide creates a localized environment with a high pH, leading to the gradual decomposition
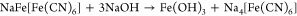
4The second step of the mechanism has been previously reported as a degradation mechanism for Prussian blue in basic media, forming [Fe(CN)_6_]^4–^ in solution. On drying and in the presence of sodium ions, Na_4_[Fe(CN)_6_] will crystallize.^45^ It should be noted that Fe(OH)_3_ was not detected by the methods reported in this study. However, Fe(OH)_3_ itself has low thermodynamic stability and decomposes to α-Fe_2_O_3_ or α-FeOOH upon drying at ∼200 °C or under alkaline conditions.^46−48^ Decomposition to iron(III) oxides matches the trends observed from XPS.

The two-step nature of the degradation has a significant implication when considering the electrochemical performance of the material. The loss of sodium in the first step (reaction 3) is essentially reversible, whereas the second step (reaction 4) is irreversible. However, Na_4_[Fe(CN)_6_] is electrochemically active with a potential ∼3.4 V vs Na/Na^+^ compared to ∼3.3 V for the low-spin iron center in PW.^36^ Thus, the degradation pathway was further confirmed by measuring the change of specific capacity in the first two cycles for Prussian white exposed to 55% RH as a function of time. The 55% RH was selected as it is closest to the humidity conditions in our lab.

### Electrochemical Degradation as a Function of Time

**Figure 7:**
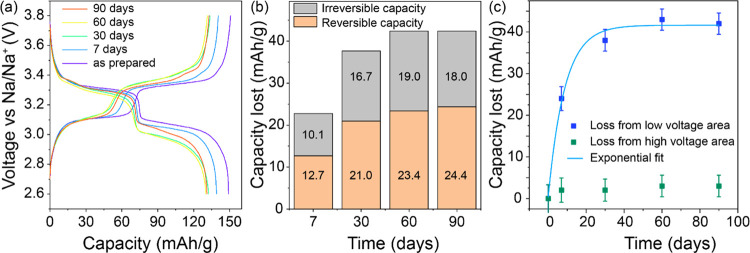
(a) Second-cycle charge/discharge voltage profiles of R-PW/Prussian blue full cell at 0.1 C. (b) Capacity lost vs storage time under 55% RH (the capacity loss values have been normalized to the capacities of the pristine electrode). (c) Capacity fade over time fitted to an exponential function. The upper cutoff voltage was limited to 3.8 V vs Na/Na^+^, to avoid any extra capacity arising from extraction of residual water.

To identify reversible and irreversible sodium loss, the specific capacities of the first and second cycles were compared to the specific capacity of a pristine sample (Figure S10). Side reactions were minimized by cycling dehydrated R-PW against a partially charged PW electrode present in excess in a voltage window of 2.6–3.8 V vs Na/Na^+^ at a rate of 0.1 C (Figure 7a). The total capacity lost was determined based on the difference in capacity in the first charge capacity relative to the as-prepared material. As sodium loss due to reaction 3 is considered reversible, this capacity will be regained during the first discharge.

Subsequently, the capacity difference between the as-prepared sample and the second charge of the exposed samples can be considered irreversible capacity loss due to reaction 4. These results are summarized in Figure 7b, where it can be seen that both reversible and irreversible capacity losses follow a similar trend, whereas the reversible losses are slightly higher. Finally, as Na_4_[Fe(CN)_6_] is electrochemically active at 3.4 V, the total losses of capacity from the high-voltage (3.3–3.6 V) and low-voltage (3.0–3.3 V) regions are compared in Figure 7c. As expected, essentially all capacity is lost from the low-voltage plateau, as the high-spin iron center will be oxidized first in reaction 3 and all low-spin iron centers lost from PW in reaction 4 are converted to Na_4_[Fe(CN)_6_]. This is further emphasized by the growth of an additional plateau at ∼3.4 V in Figure 7a. Finally, it is interesting to note that the capacity fade drops exponentially as a function of time according to
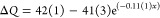
5where Δ*Q* is the loss of capacity after *x* days. The fitted parameters are *Q*_m_ = 42(1) mAh/g, the maximum capacity lost; *C* = 41(3), a scaling constant fixing Δ*Q* = 0 at *x* = 0; and *k* = 0.11(1)/day, the rate constant for capacity fade. The capacity fade was fitted according to eq 5 as it is the total capacity lost relative to an arbitrary starting capacity that is of interest. Both the rate of fade and maximum capacity lost will be of interest in future studies aimed at designing material modifications that protect against oxidative damage. Details of the fitting are provided in Table S11.

## Discussion

**Figure 8:**
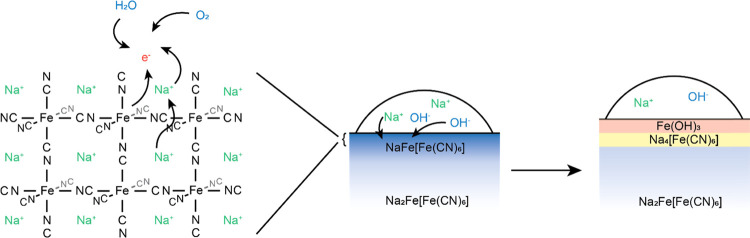
Schematic illustration of the mechanism of Na loss and formation of Na_4_[Fe(CN)_6_].

On the basis of the analysis performed as a function of time and relative humidity level, a degradation mechanism for Prussian white can be described according to Figure 8. Specifically, that sodium is leached from the bulk structure via a redox reaction involving water and oxygen to form NaOH, which subsequently leads to material decomposition forming Fe(OH)_3_ and Na_4_[Fe(CN)_6_]. This mechanism has several important consequences when considering the commercial implementation of PW. The first step is spontaneous as the oxidative power of water and oxygen together is slightly higher than that of the high-spin iron, leading to a maximum desodiation threshold of NaFe[Fe(CN)_6_], beyond which further oxidation would not be spontaneous. If in the presence of deoxygenated water- or moisture-free air, no loss of sodium would occur. However, it appears from the first part of this study that there is a critical relative humidity threshold (≥33%) beyond which significant degradation becomes observable from structure and oxidation state change as well as surface deposits after 7 days (Figures 1–5). This critical humidity threshold may explain the report by Goodenough et al.,^7^ where no significant difference in battery performance was observed after exposing a R-Na_1.92_Fe[Fe(CN)_6_] electrode in dry air for 20 h, while the same phase was lost within 25 min of air exposure for other groups.^17^ However, in these studies, the relative humidity levels were not reported. This would be an important detail to record, as according to the diffraction patterns in Figure 2, the R-PW dehydrated *R*3̅ compound experiences increased sensitivity to moisture. Specifically, the R-PW sample reverts back to a hydrated monoclinic phase under ≤6% and to a cubic phase when RH ≥ 55% after 7 days, with the latter phase transition due to a loss of sodium. Further, the reflection due to Na_4_[Fe(CN)_6_] is more intense in this sample at 75% RH, further confirming extensive loss of sodium. The phase transition between R-PW and M-PW has been demonstrated for both Na_2_Mn[Fe(CN)_6_] and Na_2_Fe[Fe(CN)]_6_ and coincides with an ∼18% volume change.^9,17^ The resulting particle cracking from the change in volume and increase of surface area is likely the origin of the higher sensitivity of R-PW relative to M-PW. Another intriguing consequence of the proposed mechanism is the role that Fe(OH)_3_/Fe_2_O_3_ and Na_4_[Fe(CN)_6_] play in passivating the material from further oxidative damage. This is reflected in the exponential decay of both reversible and irreversible capacity loss over time (Figure 7c). That is, both are prevented due to restricted access of water and oxygen. If no passivating layer were formed, reaction with oxygenated water would completely convert PW to NaFe[Fe(CN)_6_]. The effectiveness of the passivation will be dependent on factors such as relative humidity level, particle size, surface area, and surface treatment.^27^ This is partially demonstrated by the results in the first part of the study where after 7 days, a higher RH% led to more sodium loss from the bulk structure. It is also interesting to put our results in the context of recent work by Yang et al.,^27^ where surface passivation of PBAs was achieved using acetic acid to attain humidity stability. In this study, the role of acetic acid may be to remove surface oxides and hydroxides. These surface deposits would first appear following washing with deionized water. The acidified surface would modify the decomposition pathway following subsequent water exposure as eq 1 assumes neutral pH. Of course, further work would be needed to confirm this hypothesis.

Finally, the specific role that the surface deposits play during electrochemical cycling has never been directly explored before. However, preliminary results imply increased polarization in addition to the aforementioned capacity loss (Figure 7a). The origin of increased polarization is beyond the scope of this study and will need to be explored further considering the interaction with electrolyte, salt, and additives. Nevertheless, this study provides a crucial starting point on which such studies can be based. This is particularly important considering the wide variability of electrochemical performance observed for PW in the literature. Assuming that dry room conditions were not implemented, the average relative humidity in regions where a large part of PBAs work has been performed are all well above 33% RH (Table S12). Thus, this work provides a framework from which the rate and maximum capacity fade can be quantified as a function of factors such as particle size, humidity level, or surface treatments aimed at improving material stability.

## Conclusions

The effect of moisture exposure on M-PW and R-PW was investigated as a function of relative humidity and time. It was determined that the degradation occurs via a two-step mechanism, whereby the first step involves a redox reaction between PW and water and oxygen resulting in oxidation of the high-spin iron center and a loss of sodium. This step is essentially reversible without damage to the structure. The second step involves an irreversible structural decomposition under basic conditions eventually producing various iron oxides and hydroxides, as well as Na_4_[Fe(CN)_6_]. Intriguingly, these byproducts form a passivating layer preventing further material degradation over time while still allowing sodium transport in an electrochemical cell, albeit with higher surface resistance. This fundamental understanding of the processes that lead to material aging and highly variable electrochemical results provides the necessary groundwork from which strategies aimed at protecting the material can be designed and their efficacy quantified. Further, knowledge of material decomposition products is critical for understanding the undesired interactions with different electrolyte and salt combinations, which ultimately lead to cell failure. Thus, a profound knowledge on proper handling, treatment, and storage of materials is crucial in realizing better NIBs for future grid-scale electric energy storage applications.

## Supporting Information Available

Pawley fits and Rietveld refinements, infrared and Raman spectroscopy, electrochemical cycling data, SEM, and Mössbauer results (PDF)
