# Supplementary material for: Moisture-Driven Degradation Pathways in Prussian White Cathode Material for Sodium-Ion Batteries
Source: ACS Appl Mater Interfaces. 2021 Feb 18;13(8):10054–63. doi: 10.1021/acsami.0c22032 (PMC8026098; doi:10.1021/acsami.0c22032)
Supplement: Supplementary file 1 — am0c22032_si_001.pdf [file am0c22032_si_001.pdf]

SUPPORTING INFORMATION

Moisture-Driven Degradation Pathways in Prussian White Cathode Material for Sodium-Ion Batteries

Dickson O. Ojwang,*,† Mikael Svensson,† Christian Njel,‡ Ronnie Mogensen,† Ashok S. Menon,† Tore Ericsson,† Lennart Häggström,† Julia Maibach‡ and William R. Brant*,†

†Department of Chemistry – Ångström Laboratory, Uppsala University, Box 538, SE-751 21 Uppsala, Sweden

‡Institute for Applied Materials (IAM) and Karlsruhe Nano Micro Facility (KNMF), Karlsruhe Institute of Technology (KIT) Hermann-von-Helmholtz-Platz 1, 76344 Eggenstein-Leopoldshafen, Germany

*Email: dojjwa@gmail.com.

*Email: william.brant@kemi.uu.se.

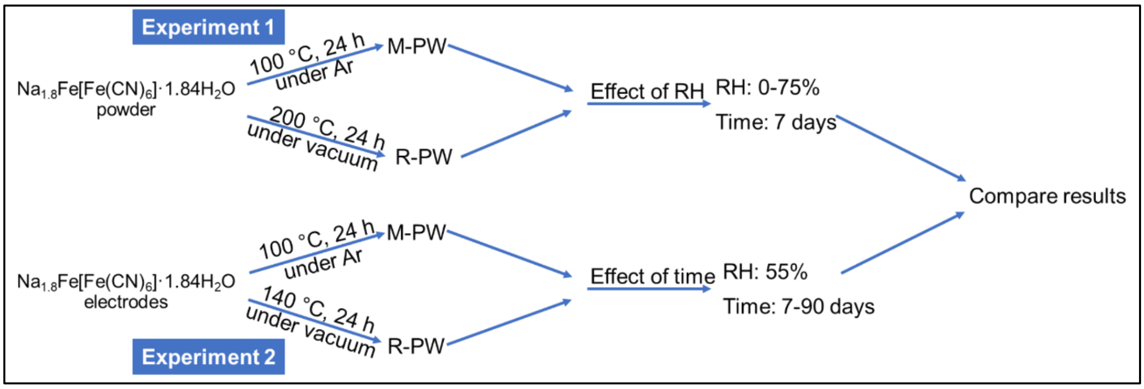

Figure S1. Schematic illustration of experimental procedure. More details on the sample composition can be found in the main manuscript.

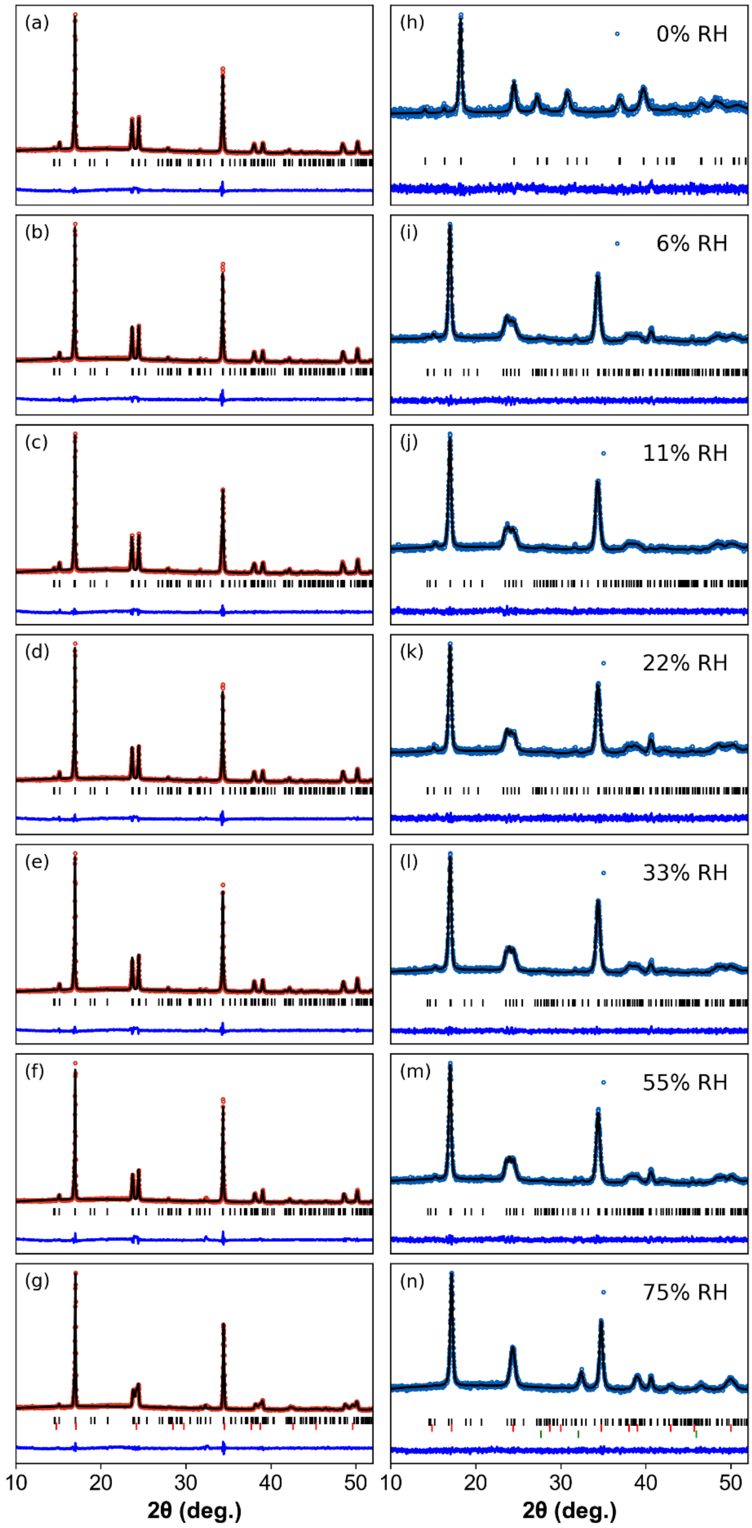

Figure S2. Pawley fits[1] of (a-g) M-PW and (h-n) R-PW structure models against powder XRD data. Observed and calculated intensities are shown as coloured circles and black lines, respectively. The difference between them is denoted by the blue line and the positions of Bragg reflection of the phases by the vertical markers. A Pawley fit of the pattern shown in (g) was done in space groups ($P2_1/n$, and $Fm\bar{3}m$) and provided satisfactory fitting procedure. The two phases together with cubic NaCl yielded a good fit of the pattern shown in (n). The unit cell parameters are given in Tables S2 and S3.

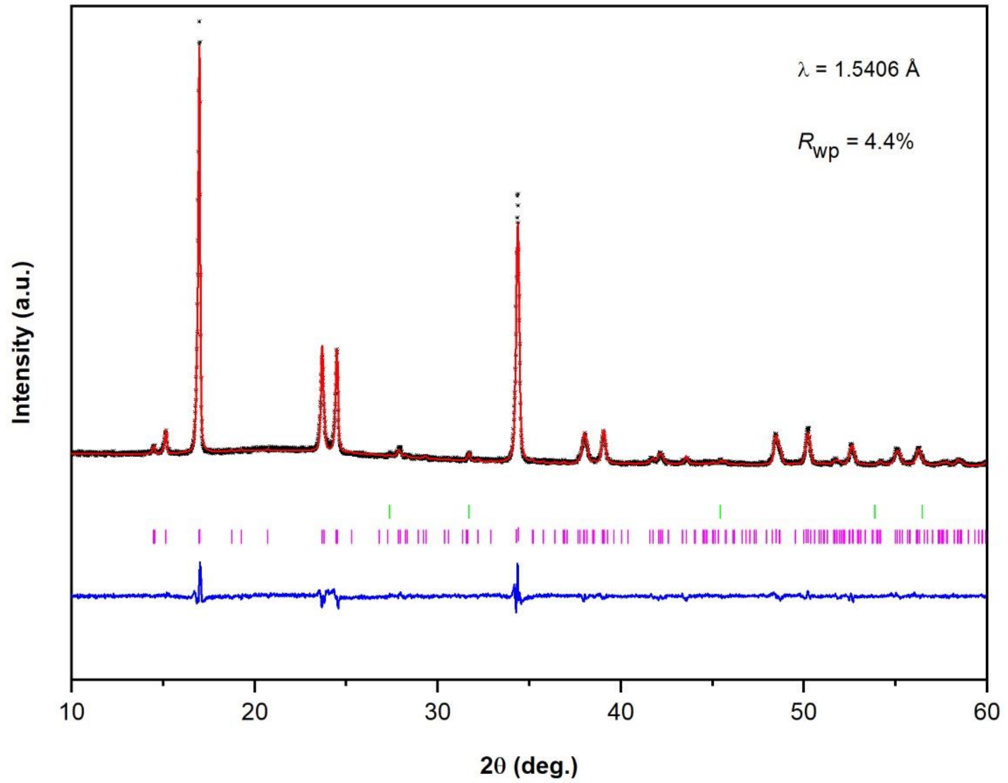

Figure S3. Rietveld refinement of XRD patterns of the as-prepared monoclinic $Na_{1.80(5)}Fe[Fe(CN)_6]_{0.95(3)}\cdot 1.84(3)H_2O$ (M-PW) sample and a cubic phase NaCl present as an impurity. The observed (black), calculated (red), and difference (blue) XRD profiles. Vertical markers show the Bragg positions (M-PW-pink, and NaCl-green). More details are provided in Tables S4 and S5.

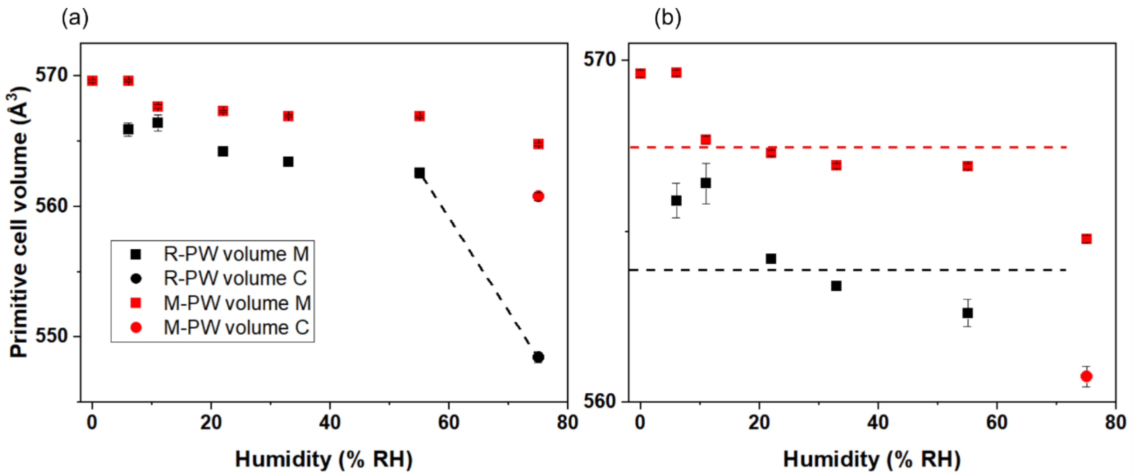

Figure S4. (a) The change of unit cell volume for the M-PW and R-PW. The cell volume for the dehydrated R-PW is not shown as it lies outside the scale drawn. (b) An enlargement of the unit cell volume data. The dashed lines are for eye guidance only. M = monoclinic, C = cubic. The equivalent volume of the cubic and rhombohedral unit cells have been transformed to corresponding monoclinic primitive cell volume.

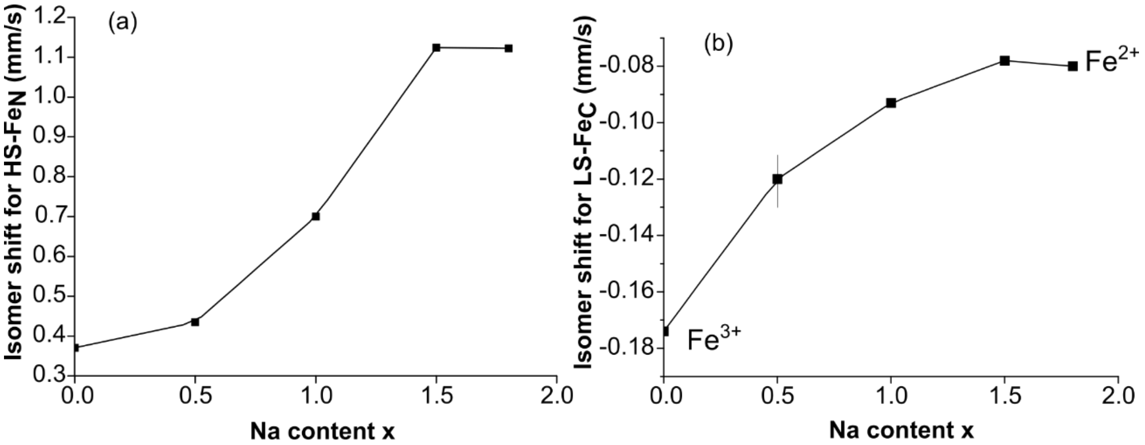

Figure S5. The average isomer shift (δ) values for (a) high-spin Fe_N and (b) low-spin Fe_C as a function of Na content x.[2,3] Each data point has an error of 5%. The lines are for eye guide only.

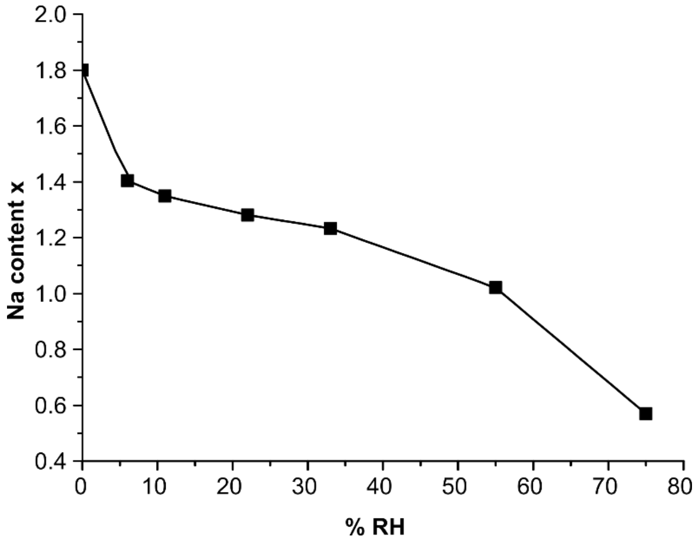

Figure S6. Na content x in monoclinic Prussian white, $Na_{1.80(5)}Fe[Fe(CN)_6]_{0.95(3)}\cdot nH_2O$ versus relative humidity (% RH). Each data point has an error of 5%. The line is for eye guide only.

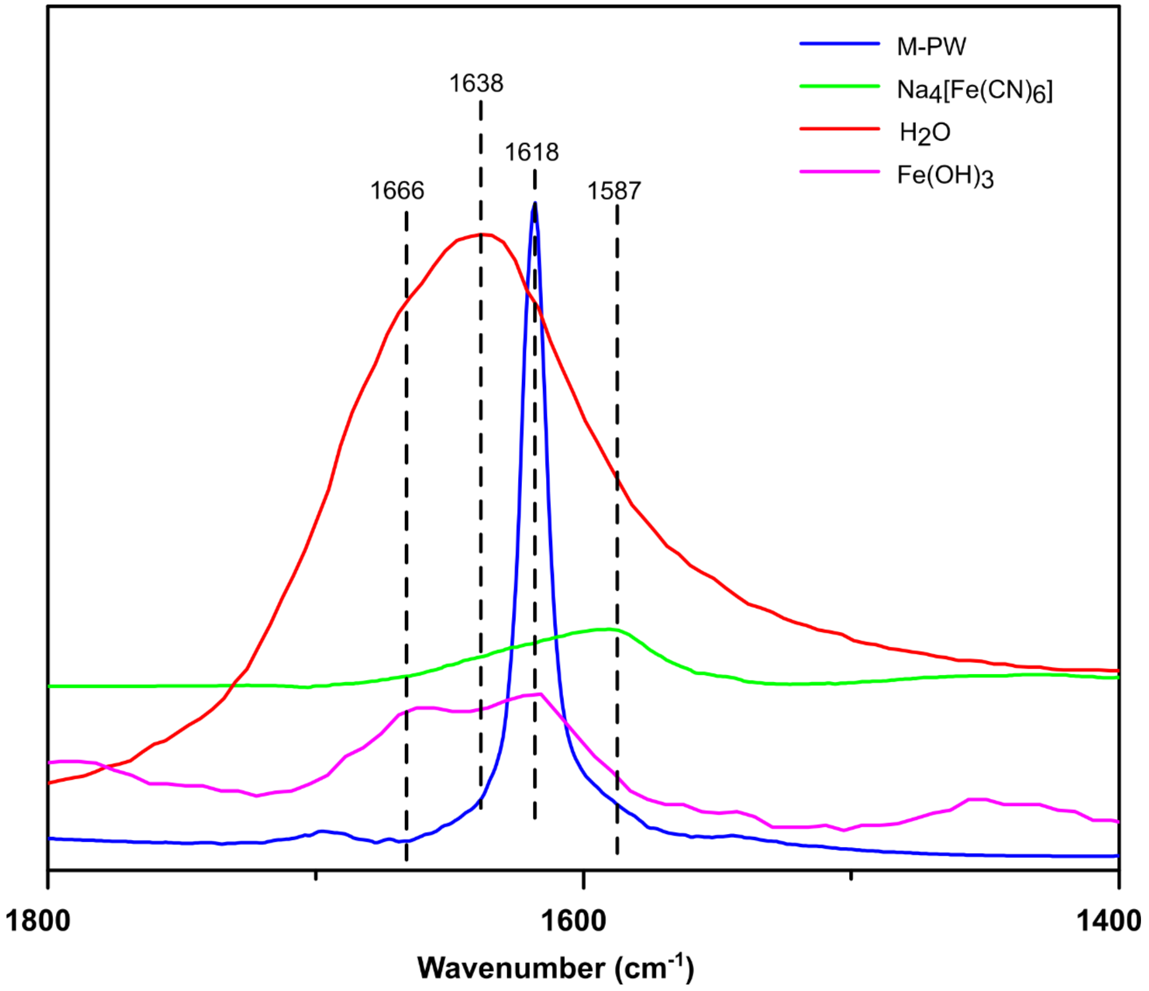

Figure S7. IR spectra of $Fe(OH)_3$, H_2O, $Na_4[Fe(CN)_6]$, and M-PW.

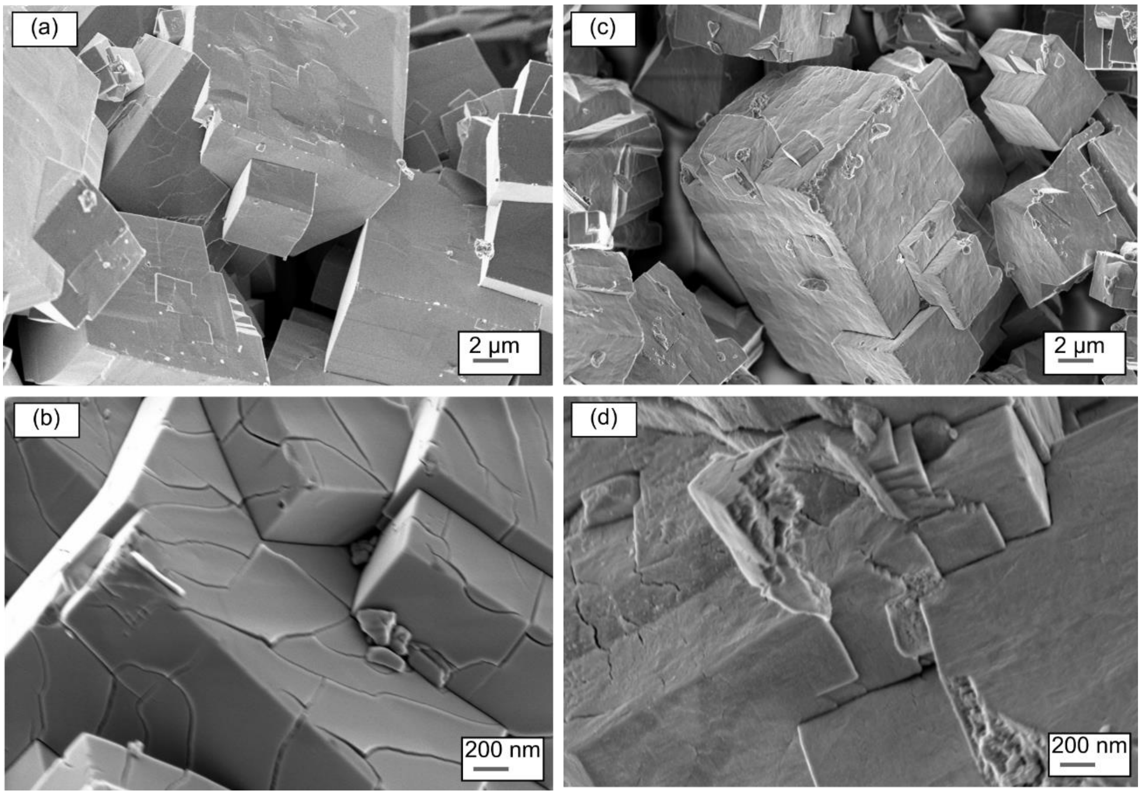

Figure S8. SEM images of (a, b) M-PW and (c, d) R-PW at 0% RH at two different magnifications obtained using an accelerating voltage of 2 kV.

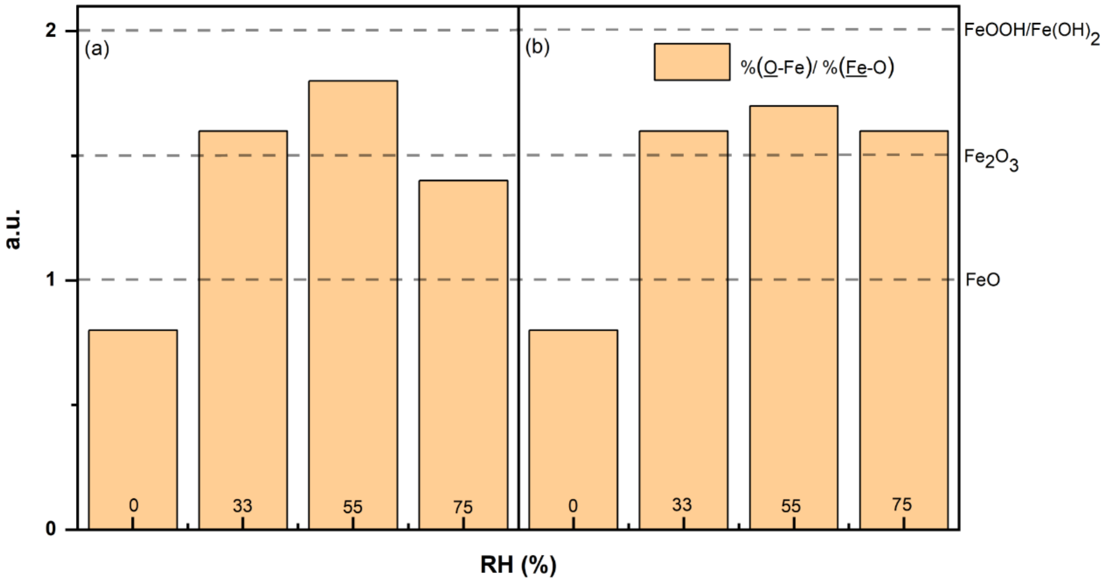

Figure S9. A comparison of %(O-Fe) to %(Fe-O) of (a) M-PW and (b) R-PW from the O 1s and Fe 2p spectra. The values on the y axis represent the ratio of Fe : O, for instance 1 implies that the valence state of iron is closer to +2, while 2 corresponds to an oxidation state closer to +3.

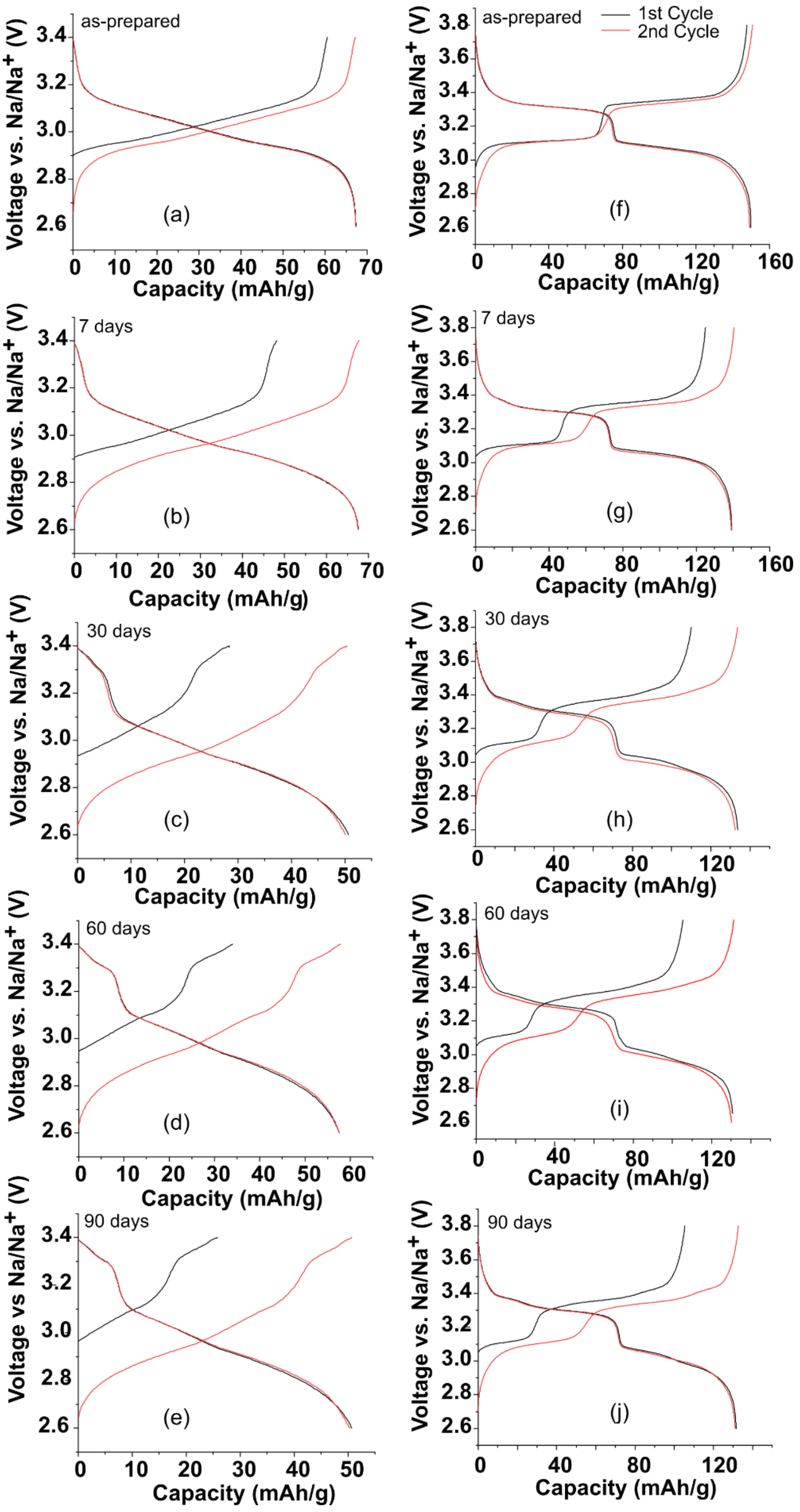

Figure S10. Galvanostatic charge-discharge profile for the 1st and 2nd cycles of (a-e) M-PW and (f-j) R-PW electrodes at 55% RH.

Table S1. Saturated aqueous salt solutions at 25 °C (±0.1°C)[4]

Salt	Humidity (% RH)
LiBr	~6
LiCl	~11
K(OAc)	~22
$MgCl_2$	~33
$Mg(NO_3)_2$	~55
NaCl	~75

Table S2. Pawley fit results for M-PW (S.G. $P2_1/n$, and $Fm\bar{3}m$)

RH (%)	Lattice parameters	Volume (Å^3)	chi^2
0	a = 10.474(1) Å, b = 7.484(1) Å, c = 7.275(1) Å, β = 92.71(1) °	569.6(1)	2.9
6	a = 10.470(1) Å, b = 7.484(1) Å, c = 7.277(1) Å, β = 92.65(1) °	569.6(1)	2.4
11	a = 10.444(1) Å, b = 7.480(1) Å, c = 7.275(1) Å, β = 92.79(1) °	567.7(1)	3.5
22	a = 10.443(1) Å, b = 7.476(1) Å, c = 7.275(1) Å, β = 92.77(1) °	567.3(1)	2.5
33	a = 10.441(1) Å, b = 7.471(1) Å, c = 7.276(1) Å, β = 92.72(1) °	566.9(1)	2.6
55	a = 10.440(1) Å, b = 7.446(1) Å, c = 7.299(1) Å, β = 92.38(1) °	566.9(1)	3.6
75	a = 10.438(1) Å, b = 7.405(1) Å, c = 7.311(1) Å, β = 92.07(1) °	564.8(1)	2.6
	$a = b = c$ = 10.390(3) Å	1121.5(3)	

Table S3. Pawley fit results for R-PW (S.G. $R\bar{3}$, $P2_1/n$, and $Fm\bar{3}m$)

RH (%)	Lattice parameters	Volume (Å^3)	chi^2
0	a = 6.539(2) Å, c = 18.878(8) Å, γ = 120 °	699.1(4)	1.1
6	a = 10.451(8) Å, b = 7.436(3) Å, c = 7.302(3) Å, β = 94.33(6) °	565.9(5)	1.4
11	a = 10.447(8) Å, b = 7.437(4) Å, c = 7.309(4) Å, β = 94.15(7) °	566.4(6)	1.3
22	a = 10.444(8) Å, b = 7.425(3) Å, c = 7.294(3) Å, β = 94.09(6) °	564.2(5)	1.1
33	a = 10.436(7) Å, b = 7.417(2) Å, c = 7.296(2) Å, β = 94.05(6) °	563.4(4)	1.4
55	a = 10.437(6) Å, b = 7.409(2) Å, c = 7.294(2) Å, β = 94.03(5) °	562.6(4)	1.3
75	$a = b = c$ = 10.313(2) Å	1096.7(6)	1.2
NaCl[5]	$a = b = c$ = 5.51(3) Å	167(3)	

Table S4. Crystal structure data for $Na_{1.80(5)}Fe[Fe(CN)_6]_{0.95(3)}\cdot 1.84(3)H_2O$, space group $P2_1/n$, a = 10.458(1) Å, b = 7.474(1) Å, and c = 7.280(1) Å; β = 92.71(1)°

atom	sites	x	y	z	occupancy
Fe1	2a	0.5	0.5	0.5	1
Fe2	2d	0.5	0	0	0.95
C1	4e	0.518(5)	0.700(7)	0.310(8)	0.95
C2	4e	0.672(4)	0.51(1)	0.500(5)	0.95
C3	4e	0.489(6)	0.684(7)	0.663(8)	0.95
N1	4e	0.515(4)	-0.187(5)	0.197(6)	0.95
N2	4e	0.281(4)	-0.003(7)	-0.006(5)	0.95
N3	4e	0.513(4)	0.205(5)	0.224(7)	0.95
Na1	4e	0.796(1)	0.544(2)	0	0.9
O1	4e	0.761(6)	0.749(9)	0.271(9)	0.5(3)
O2	4e	0.263(5)	0.708(6)	0.265(7)	0.5(3)
O3	2b	0	0	0.5	0.16(5)
O4	2c	0	0.5	0	0.36(5)
Weighted profile R-factor Rwp = 4.4%, R_{Bragg} = 1.9, χ^2 = 2.9					

Table S5. Crystal structure data for NaCl, space group $Fm\bar{3}m$, a = 5.640(1) Å

atom	sites	x	y	z	occupancy
Na1	4a	0	0	0	1
Cl1	4b	0.5	0.5	0.5	1
Weighted profile R-factor Rwp = 4.4%, R_{Bragg} = 1.9, χ^2 = 2.9					

Table S6. The results from the fitting of all Prussian white spectra. The isomer shift δ, the electric quadrupole splitting Δ and the individual Lorentzian line width Γ at FWHM are given in mm/s and the spectral intensity I in %. Experimental errors in δ, Δ and Γ are ± 0.01 mm/s and in I ± 2%, except otherwise stated. M stands for monoclinic and C for cubic crystal structures. The C coordinated Fe are in a low-spin state (LS-Fe_C), while the N coordinated Fe are in a high-spin state (HS-Fe_N). Also given is the estimated sodium content x shown in Fig. S6. Errors in x are ± 0.05

RH (%)	Na	Phase from XRD	LS-Fe_C				HS-Fe_N			
			δ	Δ	Γ	I	δ	Δ	Γ	I
0	1.80	M	-0.074(5)	0.15	0.28	50	1.12	1.09	0.39	50
6	1.41	M	-0.073(5)	0.13	0.31	50	1,11	1,09	0.38	43
							0.72	0.43	0.73	7
11	1.35	M	-0.073(5)	0.13	0.30	50	1.11	1.10	0.39	40
							0.62	0.45	0.63	10
22	1.28	M	-0.077(5)	0.12	0.32	50	1.10	1.09	0.40	36
							0.55	0.38	0.65	14
33	1.23	M	-0.079(5)	0.12	0.34	50	1.10	1.10	0.45	30
							0.64	0.24	0.84	20
55	1.02	M	-0.086(5)	0.10	0.33	50	0.95	1.26	0.63	18
							0.63	0.11	0.67	32
75	0.57	M, C	-0.097(5)	0.10	0.33	50	1.00	1.00	0.50	3
							0.45	0.40	0.48	47

Table S7. IR frequencies for M-PW

Sample	ν(O-H)	ν(CN)	δ(O-H-O)
0% RH	3646sh, 3618s, 3591sh, 3551m	2083m, 2070s, 2056sh	1618s
6% RH	3646sh, 3618s, 3591sh, 3551m	2083m, 2068s, 2050sh	1618s
11% RH	3646sh, 3618s, 3591sh, 3551m	2083m, 2069s, 2055sh	1618s
22% RH	3646sh, 3618s, 3591sh, 3551m	2084m, 2070s, 2054sh	1618s
33% RH	3646sh, 3618s, 3591sh, 3551m	2085m, 2071m, 2054w	1618s
55% RH	3646sh, 3628w, 3594w, 3558br	2084w, 2057br, 2023sh, vw	1618m
75% RH	3646sh, 3629sh, 3591m, 3566sh, 3520br, 3474vw, 3396sh, vbr	2084w, 2065w, br, 2051w, 2042vw, 2020br	1635w, 1618m

Table S8. Raman shifts for M-PW

Sample	ν(CN)
0% RH	2130s, 2093s, 2084sh, 2072sh, vbr
6% RH	2130s, 2093s, 2083sh, 2072sh, vbr
11% RH	2129s, 2106sh, vw, 2093s, 2083sh, 2071sh, vbr
22% RH	2130s, 2107sh, w, 2093s, 2084sh, 2072sh, vbr
33% RH	2131s, 2108w, 2093m, 2072sh, vbr
55% RH	2140s, 2115m, br, 2105vw, 2069sh, vbr
75% RH	2140s, 2117m, 2105vw, 2077sh, vbr

Table S9. IR frequencies for R-PW

Sample	ν(O-H)	ν(CN)	δ(O-H-O)
0% RH	-	2052s, 2023w	-
6% RH	3648w, 3622s, 3588vw, sh, 3552m	2088w, 2070s	1619s
11% RH	3648w, 3620s, 3588vw, sh, 3551m	2087w, 2066s	1618s
22% RH	3647w, 3621s, 3588vw, sh, 3552m	2081m, 2055m	1617s
33% RH	3648w, 3622s, 3588vw, sh, 3551m	2073s, 2050m	1616s
55% RH	3648vw, 3627vvw, 3597w, 3566vvw	2075m, 2049m	1612s
75% RH	3620sh, vw, 3595sh, vw, 3586m, br, 3518w, 3397vw, vbr	2065m, br, 1992vw, vbr	1631sh, 1611m

Table S10. Raman shifts for R-PW

Sample	ν(CN)
0% RH	2109m, 2068s
6% RH	2136s, br, 2105s, 2068m
11% RH	2134s, br, 2106s, 2068m
22% RH	2136s, br, 2108s, 2068m
33% RH	2142s, br, 2108s, 2068m
55% RH	2148s, br, 2102vvw, br
75% RH	2149s, br, 2096vvw, br

s: strong, m: medium, w: weak, br: broad, sh: shoulder, vw: very weak, vvw: very very weak, vbr: very broad

Table S11. Fitting parameters for capacity fade

Q_m (mAh/g)	C	*k* (/day)	R^2
42(1)	41(3)	0.11(1)	0.99

Q_m = the maximum capacity lost, C = a scaling constant fixing the loss of capacity after *x* days, and *k* = the rate of capacity fade.

Table S12. The RH values in a few selected regions[6]

Region	% RH
Singapore	~84
China (Wuhan)	~80
Sweden (Uppsala)	~75
USA (Texas, Austin)	~67
Australia (Sydney)	~57

REFERENCES

(1) Pawley, G. S. Unit-Cell Refinement From Powder Diffraction Scans. *J. Appl. Cryst.* **1981**, 14 (6), 357–361.
(2) Brant, W. R.; Mogensen, R.; Colbin, S.; Ojwang, D. O.; Schmid, S.; Häggström, L.; Ericsson, T.; Jaworski, A.; Pell, A. J.; Younesi, R. Selective Control of Composition in Prussian White for Enhanced Material Properties. *Chem. Mater.* **2019**, *31*, 7203–7211.
(3) Ojwang, D. O.; Häggström, L.; Ericsson, T.; Ångström, J.; Brant, W. R. Influence of Sodium Content on the Thermal Behavior of Low Vacancy Prussian White Cathode Material. *Dalt. Trans.* **2020**, *49*, 3570–3579.
(4) Greenspan, L. Humidity Fixed Points of Binary Saturated Aqueous Solutions. *J. Res. Natl. Bur. Stand. Physics*

Chem. **1977**, *81A* (1), 89–96.
(5) Fontana, P.; Schefer, J.; Pettit, D. Characterization of Sodium Chloride Crystals Grown in Microgravity. *J. Cryst. Growth* **2011**, *324* (1), 207–211.
(6) https://weather-and-climate.com/
